# Supplementary material for: CHD4 slides nucleosomes by decoupling entry- and exit-side DNA translocation
Source: Nat Commun. 2020 Mar 23;11:1519. doi: 10.1038/s41467-020-15183-2 (PMC7090039; doi:10.1038/s41467-020-15183-2)
Supplement: Supplementary file 1 — Supplementary Information [file 41467_2020_15183_MOESM1_ESM.pdf]

CHD4 slides nucleosomes by decoupling entry- and exit-side DNA translocation

Zhong and Paudel *et al.*

Supplementary Information

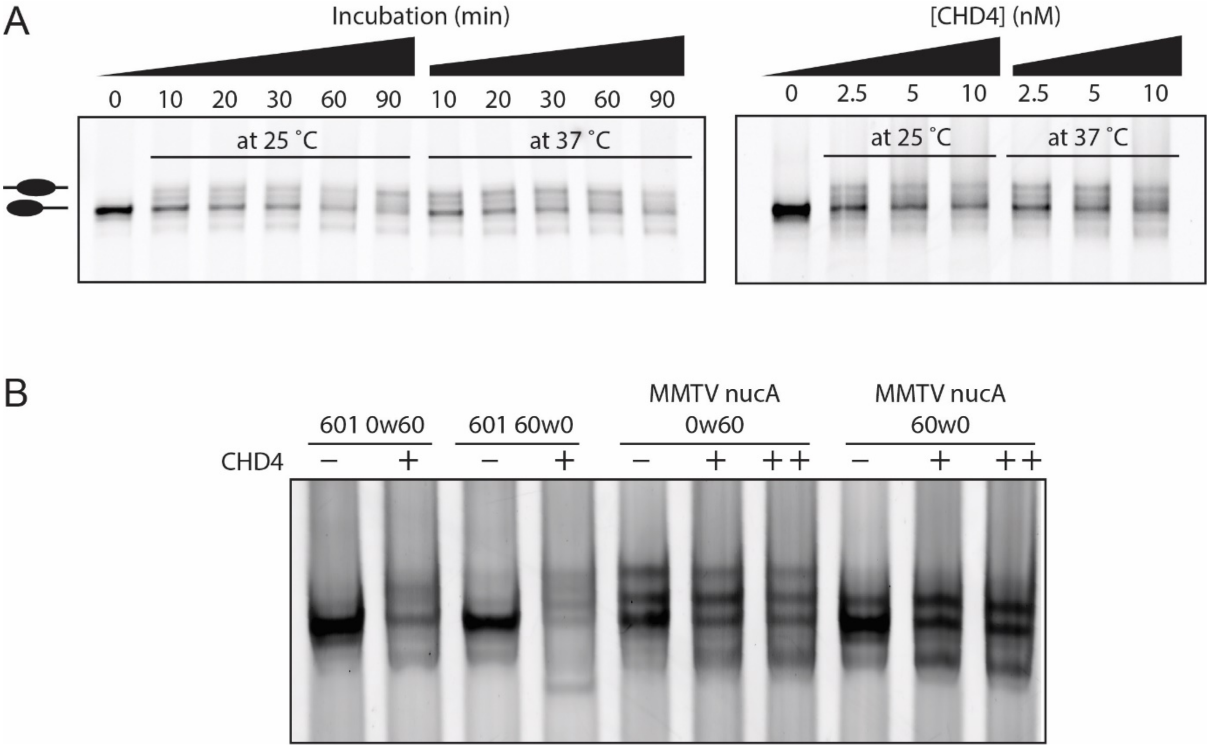

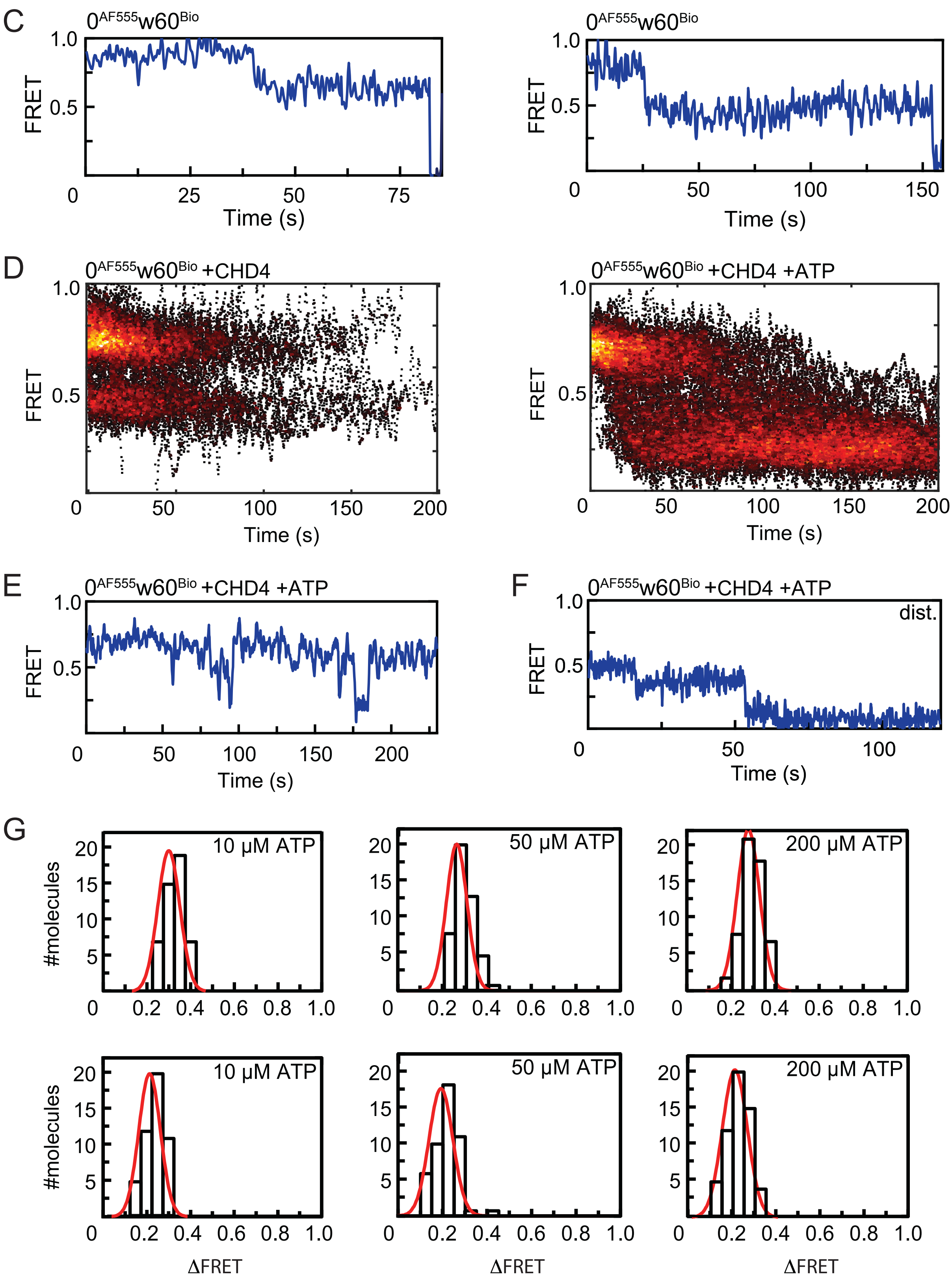

Supplementary Figure 1. Control experiments for CHD4-driven remodelling of 0w60 and $0^{AF555}w60^{Bio}$ nucleosome. A. Gel-based nucleosome repositioning assays. Time course and concentration course comparing the effect of temperature on remodelling of a 0w60 nucleosome by CHD4. The time course was carried out at a single CHD4 concentration (5 nM) and various incubation times at either 25 or 37 ˚C. The concentration course was carried out as described in Figure 1C, except the incubation temperature was either 25 or 37 ˚C.

Source data are provided as a Source Data file. **B.** Gel-based nucleosome repositioning assays carried out with the indicated nucleosomes and CHD4. 0w60 and 60w0 nucleosomes reconstituted with either 601 or MMTV nucA positioning sequences were incubated with 5 (+) or 10 (++) nM CHD4 at 37 ˚C for 60 min, the reaction was stopped by adding dsDNA (33 µg/mL) and then the samples were run on 5% native polyacrylamide gels, followed by staining in 1× SYPRO® Gold. Source data are provided as a Source Data file. **C.** FRET vs time traces of $0^{AF555}w60^{Bio}$ nucleosomes bearing both proximal and distal H2A labels. Two-step photobleaching was seen with either the distal (*left*) or the proximal (*right*) fluorophore being photobleached first. **D.** Heatmap of both proximally and distally labelled $0^{AF555}w60^{Bio}$ nucleosomes showing the time dependence of FRET upon incubation with either CHD4 alone (*left panel*, N = 105) or with 2 nM CHD4 in the presence of 1 mM ATP (*right panel*, N = 79). **E.** FRET vs time trace for $0^{AF555}w60^{Bio}$, showing the transient fluctuations that were observed occasionally in the presence of both CHD4 and ATP. **F.** FRET vs time trace for a distally labelled $0^{AF555}w60^{Bio}$ nucleosome, showing a step-wise decrease when incubated with 1 mM ATP and 2 nM CHD4. **G.** Distribution of the 1st (*top*) and 2nd (*bottom*) FRET step sizes for >40 molecules undergoing remodelling in presence of 2 nM CHD4 and various concentrations of ATP. The histograms are fitted to a Gaussian distribution.

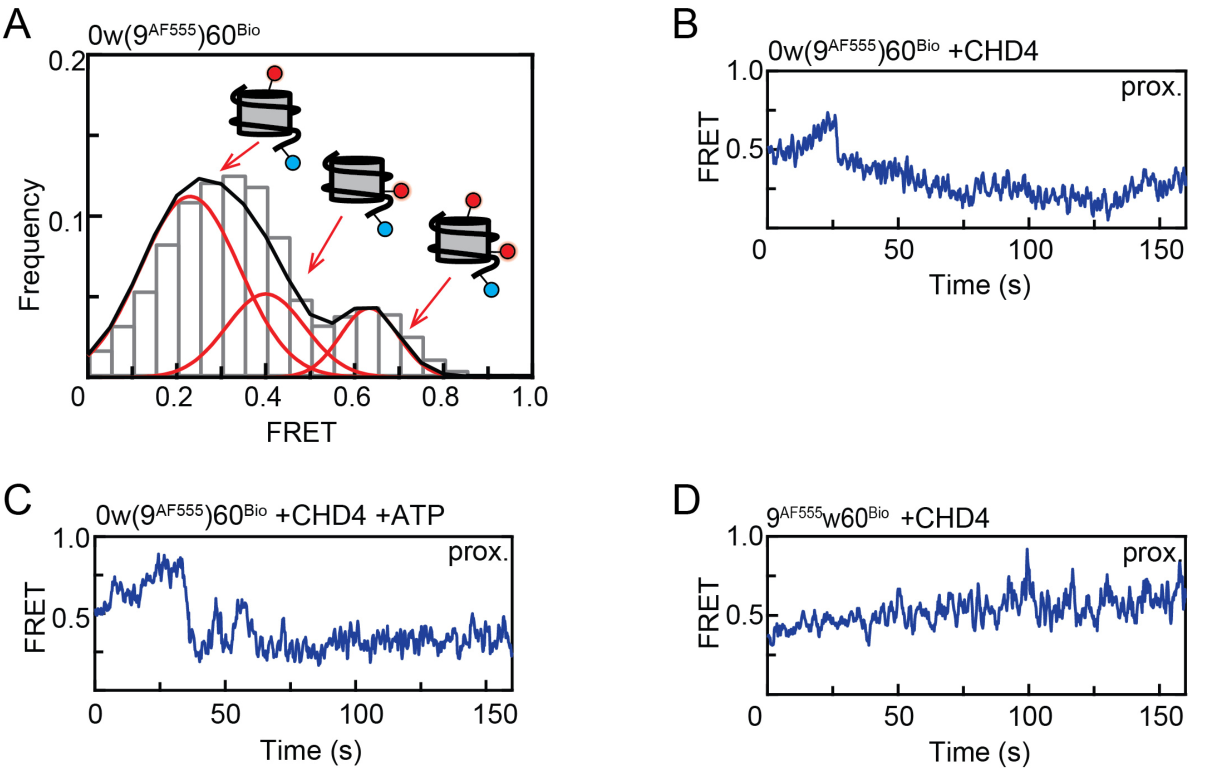

Supplementary Figure 2. Pre-reaction distribution of nucleosomal FRET states for 0w(9^{AF555})60^{Bio} nucleosomes and FRET traces of proximally labelled 0w(9^{AF555})60^{Bio} and 9^{AF555}w60^{Bio} nucleosomes undergoing CHD4 binding or remodelling. A. FRET distribution for 0w(9^{AF555})60^{Bio} nucleosomes (N = 97). The peak FRET values 0.2, 0.4, and 0.65 (obtained from a Gaussian fit, black line) correspond to particles bearing an AF647 tag at proximal, distal or both H2A subunits. **B**. FRET trace of 0w(9^{AF555})60^{Bio} bearing a proximal AF647 label, showing a gradual increase and then decrease upon binding of CHD4 (2 nM). The high FRET state is transient and quickly drops back to 0.4 and then subsequently 0.2. **C.** FRET vs time trace for 0w(9^{AF555})60^{Bio} bearing a proximal AF647 label, in the presence of both CHD4 (2 nM) and ATP (10 µM). **D.** FRET vs time trace for 9^{AF555}w60^{Bio} bearing a proximal AF647 label, in the presence of CHD4 (2 nM). A gradual increase is observed over time.

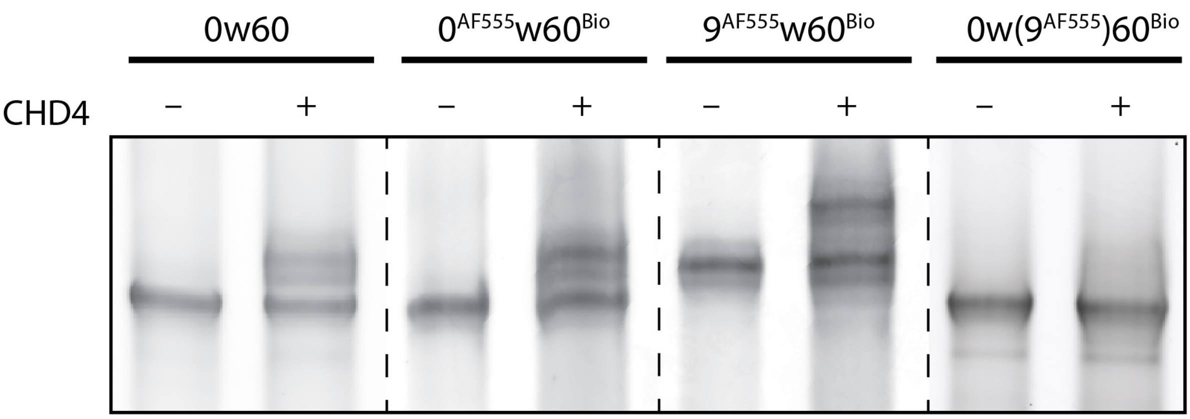

Supplementary Figure 3. Gel-based nucleosome repositioning assays for the nucleosomes used in smFRET assays. Nucleosomes containing both AF555 on DNA and

AF647 on H2A were remodelled by 10 nM CHD4 in the presence of 1 mM ATP. A positive control of unlabelled 0w60 was included. Source data are provided as a Source Data file.

```
CHD4_HUMAN LHPYQMEGLNWLRFSWAQGTDTILADEMGLGKTVQTAVFLYSLYKEGHSKGPFLVSAPLS 785
CHD1_YEAST LRDFQLTGINWMAFLWSKGDNGILADEMGLGKTVQTVAFISWLIFARRQNGPHIIVVPLS 435
CHD1_HUMAN LRDYQLNGLNWLAHSWCKGNSCILADEMGLGKTIQTISFLNYLFHEHQLYGPFLLVVPLS 540

CHD4_HUMAN TIINWEREFEMWAPDMYVVTYVGDKDSRAIIRENEFSFEDNAIRGGKKASRMKKEASVKF 845
CHD1_YEAST TMPAWLDTFEKWAPDLNCICYMGNQKSRDTIREYEFYTNPR----------AKGKKTMKF 485
CHD1_HUMAN TLTSWQREIQTWASQMNAVVYLGDINSRNMIRTHEWTHH--------------QTKRLKF 586

CHD4_HUMAN HVLLTSYELITIDMAILGSIDWACLIVDEAHRLKNNQSKFFRVLNGYSLQHKLLLTGTPL 905
CHD1_YEAST NVLLTTYEYILKDRAELGSIKWQFMAVDEAHRLKNAESSLYESLNSFKVANRMLITGTPL 545
CHD1_HUMAN NILLTTYEILLKDKAFLGGLNWAFIGVDEAHRLKNDDSLLYKTLIDFKSNHRLLITGTPL 646

CHD4_HUMAN QNNLEELFHLLNFLTPERFHNLEGFLEEFADIAKEDQIKKLHDMLGPHMLRRLKADVFKN 965
CHD1_YEAST QNNIKELAALVNFLMPGRFTIDQEIDFENQDEEQEEYIHDLHRRIQPFILRRLKKDVEKS 605
CHD1_HUMAN QNSLKELWSLLHFIMPEKFSSWEDFEEEHGKG-REYGYASLHKELEPFLLRRVKKDVEKS 705

CHD4_HUMAN MPSKTELIVRVELSPMQKKYYKYILTRNFEALNARGGGNQVSLLNVVMDLKKCCNHPYLF 1025
CHD1_YEAST LPSKTERILRVELSDVQTEYYKNILTKNYSALTAGAKGGHFSLLNIMNELKKASNHPYLF 665
CHD1_HUMAN LPAKVEQILRMEMSALQKQYYKWILTRNYKALSKGSKGSTSGFLNIMMELKKCCNHCYLI 765

CHD4_HUMAN PVAAMEAP-KMPNGMY----DGSALIRASGKLLLLQKMLKNLKEGGHRVLIFSQMTKMLD 1080
CHD1_YEAST DNAEERVLQKFGDGKMTRENVLRGLIMSSGKMVLLDQLLTRLKKDGHRVLIFSQMVRMLD 725
CHD1_HUMAN KPPDNNEF-------YNKQEALQHLIRSSGKLILLDKLLIRLRERGNRVLIFSQMVRMLD 818

CHD4_HUMAN LLEDFLEHEGYKYERIDGGITGNMRQEAIDRFNAPGAQQFCFLLSTRAGGLGINLATADT 1140
CHD1_YEAST ILGDYLSIKGINFQRLDGTVPSAQRRISIDHFNSPDSNDFVFLLSTRAGGLGINLMTADT 785
CHD1_HUMAN ILAEYLKYRQFPFQRLDGSIKGELRKQALDHFNAEGSEDFCFLLSTRAGGLGINLASADT 878

CHD4_HUMAN VIIYDSDWNPHNDIQAFSRAHRIGQNKKVMIYRFVTRASVEERITQVAKKKMMLTHLVVR 1200
CHD1_YEAST VVIFDSDWNPQADLQAMARAHRIGQKNHVMVYRLVSKDTVEEEVLERARKKMILEYAIIS 845
CHD1_HUMAN VVIFDSDWNPQNDLQAQARAHRIGQKKQVNIYRLVTKGSVEEDILERAKKKMVLDHLVIQ 938
```

Supplementary Figure 4. Sequence alignment of the ATPase domain of human CHD4 with human and yeast CHD1. Of the 19 residues that make contact with the DNA in the structure of CHD1 bound to the nucleosome (PDB: 5O9G, (Farnung et al., 2017), marked in *yellow*), conserved residues are highlighted by *black boxes*. Additional conservation between human CHD1 and CHD4 is highlighted by *red boxes*.
