# Supplementary material for: CHD4 slides nucleosomes by decoupling entry- and exit-side DNA translocation
Source: Nat Commun. 2020 Mar 23;11:1519. doi: 10.1038/s41467-020-15183-2 (PMC7090039; doi:10.1038/s41467-020-15183-2)
Supplement: Supplementary file 3 — Description of Additional Supplementary Files [file 41467_2020_15183_MOESM3_ESM.pdf]

Description of Additional Supplementary Files

File Name: Supplementary Movie 1

Description: 3D animation showing the proposed nucleosome remodelling mechanism of CHD4.
