# Supplementary material for: CHD4 slides nucleosomes by decoupling entry- and exit-side DNA translocation
Source: Nat Commun. 2020 Mar 23;11:1519. doi: 10.1038/s41467-020-15183-2 (PMC7090039; doi:10.1038/s41467-020-15183-2)
Supplement: Supplementary file 5 — Reporting Summary [file 41467_2020_15183_MOESM5_ESM.pdf]

nature research

Corresponding author(s): joel.mackay@sydney.edu.au
vanoijen@uow.edu.au

Last updated by author(s): Jul 1, 2019

# Reporting Summary

Nature Research wishes to improve the reproducibility of the work that we publish. This form provides structure for consistency and transparency in reporting. For further information on Nature Research policies, see Authors & Referees and the Editorial Policy Checklist.

## Statistics

For all statistical analyses, confirm that the following items are present in the figure legend, table legend, main text, or Methods section.

| n/a | Confirmed | |
|---|---|---|
| ☐ | ☒ | The exact sample size (*n*) for each experimental group/condition, given as a discrete number and unit of measurement |
| ☐ | ☒ | A statement on whether measurements were taken from distinct samples or whether the same sample was measured repeatedly |
| ☒ | ☐ | The statistical test(s) used AND whether they are one- or two-sided<br>*Only common tests should be described solely by name; describe more complex techniques in the Methods section.* |
| ☒ | ☐ | A description of all covariates tested |
| ☒ | ☐ | A description of any assumptions or corrections, such as tests of normality and adjustment for multiple comparisons |
| ☒ | ☐ | A full description of the statistical parameters including central tendency (e.g. means) or other basic estimates (e.g. regression coefficient) AND variation (e.g. standard deviation) or associated estimates of uncertainty (e.g. confidence intervals) |
| ☒ | ☐ | For null hypothesis testing, the test statistic (e.g. *F*, *t*, *r*) with confidence intervals, effect sizes, degrees of freedom and *P* value noted<br>*Give P values as exact values whenever suitable.* |
| ☒ | ☐ | For Bayesian analysis, information on the choice of priors and Markov chain Monte Carlo settings |
| ☒ | ☐ | For hierarchical and complex designs, identification of the appropriate level for tests and full reporting of outcomes |
| ☒ | ☐ | Estimates of effect sizes (e.g. Cohen's *d*, Pearson's *r*), indicating how they were calculated |

*Our web collection on statistics for biologists contains articles on many of the points above.*

## Software and code

Policy information about availability of computer code

| | |
|---|---|
| Data collection | Hamamatsu EMCCD camera was used to collect single-molecule movies with their provided software and micromanager 2. beta version platform was used to control camera and shutter during experiments. |
| Data analysis | Single-molecule intensity time trajectories were generated in interactive data language (IDL 8.5) and these trajectories were analyzed in MATLAB using scripts available at https://cplc.illinois.edu/software/ |

For manuscripts utilizing custom algorithms or software that are central to the research but not yet described in published literature, software must be made available to editors/reviewers. We strongly encourage code deposition in a community repository (e.g. GitHub). See the Nature Research guidelines for submitting code & software for further information.

## Data

Policy information about availability of data

All manuscripts must include a data availability statement. This statement should provide the following information, where applicable:
- Accession codes, unique identifiers, or web links for publicly available datasets
- A list of figures that have associated raw data
- A description of any restrictions on data availability

All the raw intensity time traces for Figure 3, 4, 5 and 6 and supplementary figure S1 and S2 will be deposited according Nature Communications policy

# Field-specific reporting

Please select the one below that is the best fit for your research. If you are not sure, read the appropriate sections before making your selection.

☒ Life sciences ☐ Behavioural & social sciences ☐ Ecological, evolutionary & environmental sciences

For a reference copy of the document with all sections, see nature.com/documents/nr-reporting-summary-flat.pdf

# Life sciences study design

All studies must disclose on these points even when the disclosure is negative.

| | |
|---|---|
| Sample size | Sample size was not pre-determined for single molecule assays. Instead, the number of samples required is dictated by the statistical confidence (and experimental design) with which difference between the different FRET states can be identified. In our case, the values of the FRET states can be identified unambiguously by collecting around 70-100 molecules in each conditions tested here. The sample sizes of gel-based assays were predetermined by a number of screening assays to ensure the results would cover enough shifting events. |
| Data exclusions | No data excluded. |
| Replication | 4-6 independent single molecule experiments were carried out to collect enough molecules for our analysis. Each gel-based assay has been repeated at least 3 times to test reproductivity. |
| Randomization | For single molecule assays, molecules were categorized into different FRET states by using a threshold cut off. There is no randomization for gel-based remodelling assays and EMSAs. |
| Blinding | Blinding is not possible, since molecules are identified using following criteria (1) Photobleaching of dyes or at least acceptor dye must photobleach (2) Anti-correlation change in intensity traces of donor and acceptor and (3) No blinking of dyes must occur. Hence, molecules were analysed manually. |

# Reporting for specific materials, systems and methods

We require information from authors about some types of materials, experimental systems and methods used in many studies. Here, indicate whether each material, system or method listed is relevant to your study. If you are not sure if a list item applies to your research, read the appropriate section before selecting a response.

## Materials & experimental systems

| n/a | Involved in the study | |
|---|---|---|
| ☐ | ☒ | Antibodies |
| ☐ | ☒ | Eukaryotic cell lines |
| ☒ | ☐ | Palaeontology |
| ☒ | ☐ | Animals and other organisms |
| ☒ | ☐ | Human research participants |
| ☒ | ☐ | Clinical data |

## Methods

| n/a | Involved in the study | |
|---|---|---|
| ☒ | ☐ | ChIP-seq |
| ☒ | ☐ | Flow cytometry |
| ☒ | ☐ | MRI-based neuroimaging |

## Antibodies

| | |
|---|---|
| Antibodies used | Anti-Flag Affinity Gel from Biomake.com; Cat.No. 23102; Clone name: 1E6 Mouse IgG2b; Lot No. 710037. |
| Validation | None. |

## Eukaryotic cell lines

Policy information about cell lines

| | |
|---|---|
| Cell line source(s) | Expi293 |
| Authentication | Not tested |
| Mycoplasma contamination | Not tested |
| Commonly misidentified lines (See ICLAC register) | None |
